# Widespread fMRI BOLD Signal Overactivations during Cognitive Control in Older Adults Are Not Matched by Corresponding Increases in fPET Glucose Metabolism

**DOI:** 10.1523/JNEUROSCI.1331-22.2023

**Published:** 2023-04-05

**Authors:** Lars Stiernman, Filip Grill, Charlotte McNulty, Philip Bahrd, Vania Panes Lundmark, Jan Axelsson, Alireza Salami, Anna Rieckmann

**Affiliations:** ^1^Department of Integrative Medical Biology, Umeå University, 901 87 Umeå, Sweden; ^2^Umeå Center for Functional Brain Imaging, Umeå University, 901 87 Umeå, Sweden; ^3^Department of Radiation Sciences, Umeå University, 901 87 Umeå, Sweden; ^4^Wallenberg Centre for Molecular Medicine, Umeå University, 901 87 Umeå, Sweden; ^5^Aging Research Center, Karolinska Institutet & Stockholm University, Stockholm, 171 65 Solna, Sweden; ^6^Munich Center for the Economics of Aging, Max Planck Institute for Social Law and Social Policy, Munich, 80799 München, Germany

**Keywords:** aging, fMRI, glucose metabolism, overactivation, PET, working memory

## Abstract

A common observation in fMRI studies using the BOLD signal is that older adults, compared with young adults, show overactivations, particularly during less demanding tasks. The neuronal underpinnings of such overactivations are not known, but a dominant view is that they are compensatory in nature and involve recruitment of additional neural resources. We scanned 23 young (20-37 years) and 34 older (65-86 years) healthy human adults of both sexes with hybrid positron emission tomography/MRI. The radioligand [18F]fluoro-deoxyglucose was used to assess dynamic changes in glucose metabolism as a marker of task-dependent synaptic activity, along with simultaneous fMRI BOLD imaging. Participants performed two verbal working memory (WM) tasks: one involving maintenance (easy) and one requiring manipulation (difficult) of information in WM. Converging activations to the WM tasks versus rest were observed for both imaging modalities and age groups in attentional, control, and sensorimotor networks. Upregulation of activity to WM-demand, comparing the more difficult to the easier task, also converged between both modalities and age groups. For regions in which older adults showed task-dependent BOLD overactivations compared with the young adults, no corresponding increases in glucose metabolism were found. To conclude, findings from the current study show that task-induced changes in the BOLD signal and synaptic activity as measured by glucose metabolism generally converge, but overactivations observed with fMRI in older adults are not coupled with increased synaptic activity, which suggests that these overactivations are not neuronal in origin.

**SIGNIFICANCE STATEMENT** Findings of increased fMRI activations in older compared with younger adults have been suggested to reflect increased use of neuronal resources to cope with reduced brain function. The physiological underpinnings of such compensatory processes are poorly understood, however, and rest on the assumption that vascular signals accurately reflect neuronal activity. Comparing fMRI and simultaneously acquired functional positron emission tomography as an alternative index of synaptic activity, we show that age-related overactivations do not appear to be neuronal in origin. This result is important because mechanisms underlying compensatory processes in aging are potential targets for interventions aiming to prevent age-related cognitive decline.

## Introduction

Three decades of fMRI have informed our understanding of how younger and older adults differ in brain activity, and how these differences relate to age-related differences in cognitive performance. Age-related performance deficits in taxing cognitive tasks are often accompanied by reduced brain activity in task-relevant regions (e.g., Mattay et al., 2006; Nagel et al., 2009). However, when older adults are able to perform a task well, either because the task is less taxing or because subgroups of older individuals exhibit spared cognitive capacity, fMRI studies often report increased and additional activations for older adults (e.g., Schneider-Garces et al., 2010; Spreng et al., 2010; Chen et al., 2022). The physiological underpinnings of these overactivations in aging are not understood but may reflect compensatory neural activity where additional resources are expended to maintain cognitive performance (Cabeza, 2002; Reuter-Lorenz and Cappell, 2008; Cabeza et al., 2018). However, overactivations in aging in relation to maintained cognitive performance are so far not reproducible in longitudinal settings when older adults are compared with themselves over time (Nyberg et al., 2010; Hakun et al., 2015; Rieckmann et al., 2017; Pudas et al., 2018; Vaqué-Alcázar et al., 2020), calling into question whether cross-sectional overactivations are neuronal in origin. It is of critical importance to understand the physiological basis of overactivations because compensatory mechanisms are a promising target for interventions that delay or revert cognitive decline in aging.

The fMRI BOLD signal reflects a complex interplay between metabolic demands and hemodynamics (Vazquez et al., 2018; Howarth et al., 2021), and neurovascular coupling may undergo changes with age (D'Esposito et al., 2003; West et al., 2019). The vasculature itself undergoes changes in aging (Lu et al., 2011; Tsvetanov et al., 2015; Wright and Wise, 2018), as well as the physiological determinants of neurovascular coupling, such as neurotransmitter tone (Bekar et al., 2012; Zaldivar et al., 2014) and astrocyte function (Boisvert et al., 2018; Gu et al., 2018). For this reason, it is difficult to conclude from fMRI alone whether age-related overactivations are reflective of an increased expenditure of neural resources.

In the current study, we acquired fMRI BOLD data, along with simultaneously acquired functional PET (fPET), in young and old adults during two working memory (WM) tasks. Functional PET uses dynamically acquired [^18^F]fluorodeoxyglucose (FDG) data to measure changes in glucose metabolism in active neuronal cells in response to functional task changes at the minute scale (Villien et al., 2014). fPET thus serves as a surrogate marker of synaptic activity, which, as opposed to the BOLD signal, is independent from oxygenation and blood flow (Villien et al., 2014). Prior work in young adults has demonstrated dynamic fPET signal changes in task-specific regions, indicative of increases in neuronal activity (Villien et al., 2014; Hahn et al., 2016, 2020; Jamadar et al., 2019; Ripp et al., 2021; Stiernman et al., 2021). Our own work, Stiernman et al. (2021) acquired fPET at the same time as fMRI in a hybrid scanner during WM and found remarkably close overlap between BOLD signal activations and metabolic activity in attentional and control networks in young adults. The aim of the current study was to build on this work, extend it to a sample of older adults, and test the hypothesis that fMRI overactivations in older adults are reflective of increased use of neuronal resources, as indexed by fPET (Reuter-Lorenz and Cappell, 2008; Cabeza et al., 2018; Stern et al., 2019). Critically, if overactivations in older compared with younger adults reflect a compensatory neural response, we expect to observe concomitantly increased local metabolic demand. Alternatively, overactivations may reflect vascular, non-neuronal signal changes if not accompanied by increased metabolic demand.

## Materials and Methods

### Participants

Healthy, young adults between 20 and 40 years of age and healthy, older adults above 65 were recruited via ads placed at Umeå University and a local newspaper. Participants without normal or corrected-to-normal vision, MRI incompatibility, history of head trauma, current or past diagnosis of a neurologic or psychiatric illness, drug or alcohol abuse or dependence, and use of psychopharmaceuticals, drugs, or stimulants other than caffeine or nicotine for the past 6 months were excluded. Persons that previously had undergone PET scanning for research purposes, and pregnant or breastfeeding women, were excluded because of radiation safety procedures. Five young and five older participants were excluded after recruitment because of technical problems with imaging acquisition or poor data quality, resulting in a final sample size of 23 healthy, young adults (mean age = 25.2, SD = 4.0, range = 20-37, 56.5% female) and 34 older adults (mean age = 71.09, SD = 6.03, range = 65-86, 50% female). The study was approved by the Regional Ethics Committee at Umeå University.

### Procedure

The current study is based on the same data collection and procedure as described by Stiernman et al. (2021), which presented results from young adults. In contrast to this prior work, we here compare the young adults to a group of older adults and focus on the group differences. Upon arrival, participants were informed about the study, signed the informed consent form, and then practiced the in-scanner task. Participants were asked to fast for 4 h before scanning. Blood glucose levels were measured to confirm that levels were <10 mmol/l. An intravenous needle used for infusion was then placed in the left arm. A 60 min scan included acquisition of fPET and fMRI data during verbal WM tasks, described below. At a separate occasion, participants also performed a large battery of offline cognitive tasks, described in detail by Nevalainen et al. (2015). Results from three of the offline tasks entered the analyses of the current study and are described below.

### Verbal WM tasks

During the scan, two verbal WM tasks and rest with eyes open were performed in blocks of 6 min each in the order: rest, manipulation, maintenance, rest, maintenance, manipulation, rest, for a total of 42 min. The easy condition involved simple maintenance of letters over a brief delay period, and the difficult condition involved manipulation of letters. For the maintenance condition, four target letters were presented in the center of a screen for 2 s. After a brief delay period of 3.5 s showing a fixation cross, a probe letter appeared on screen for 2.5 s and participants had to respond with their right index finger if the probe matched one of the target letters, and their right middle finger if it did not match. After 1 s, showing a fixation cross the next trial commenced. Each 6 min, block embedded six 1 min task blocks in which five trials were performed (45 s) followed by 15 s of resting fixation. This embedded design was chosen to accommodate the slow temporal resolution of PET at the minute-scale, while allowing for the analysis of fMRI data within slow blocks in terms of a traditional block design (compare Stiernman et al., 2021). The manipulation condition had the same task structure as the maintenance condition but involved only two target letters presented for 2 s. Participants had to manipulate the letters in WM into the subsequent letter in the alphabet during the 3.5 s delay period, then respond to the subsequent probe displayed for 2.5 s with their right index finger if the probe matched one of the target letters, or their right middle finger if it did not match. Across the entire experiment, accuracy was computed as percentage correct, where 100% would correspond to 60 correct trials for the given condition. Of the 60 trials, 30 trials were targets, and 30 trials were lures.

### Offline WM tasks

In order to derive a potentially more robust measure of WM function, compared with the in-scanner tasks alone, we computed a standardized unit-weighted average of three WM tasks (letter updating, numerical 3-back, and spatial updating) used previously (Nevalainen et al., 2015; Nordin et al., 2022). In other words, each task was *z*-scored individually, and the average of the three tasks formed the WM composite. Briefly, letter updating involved memorizing and updating a sequence of letters, A-D, presented randomly and sequentially at an interval of 1.5 s. At different intervals, 4 times per sequence length (7, 9, 11, 13), subjects had to type in the last three presented letters. The dependent measure was the percentage correct of a total of 48. In the columnized numerical 3-back task, numbers appeared in a 1 × 3 grid moving from left to right at an interval of 1.5 s. Subjects had to indicate whether the number presented in a box was the same or a different number than the number previously presented in that box. Four trials consisting of 30 number presentations were performed. The dependent measure was the percentage correct of a total of 120. Three subjects failed to comply to n-back instructions; hence, their average was computed as the mean of letter and spatial updating. In the spatial updating task, three 3 × 3 grids were presented. Each trial began with a circle appearing for 4 s at a random location in each of the three grids, after which they disappeared. Subsequently, an arrow appeared below one of the grids for 2.5 s, and subjects were required to update the location of the circle. Two updating operations per grid were required on every trial and subjects responded by clicking the position of the circle in each of the three grids after the six updating operations. Ten trials were performed, and the dependent measure was the percent correct of a total of 30.

The composite WM score of the older participants in the current study was compared with the performance of a sample of age- and education-matched individuals drawn from the population (Nordin et al., 2022). Bins of age groups (65-69, 70-74, and >75), and of high (≥13) and low (<13) years of education were produced, and the WM composite was computed with respect to their age- and education-matched peers' performance. In other words, *z* scoring was computed based on the means and SDs derived from the respective age- and education-matched groups drawn from the population (in total six specific means and SDs). Each task was *z*-scored individually and averaged to form the WM composite. The outcome *z* score reflects the WM function in the older participants in the current study compared with the population average, where a score of 1 would indicate that the current sample performed 1 SD above the population mean. One-sample *t* tests were used to see if the mean differed from zero. The reliability of the tasks forming the WM composite are all high, range 0.78-0.95 (Nordin et al., 2022). The means ± SDs in the comparison sample were as follows: letter updating task, 66.98 ± 5.94%; n-back task, 57.25 ± 5.04%; and spatial updating task, 33.93 ± 4.23%.

### PET/MRI acquisition and analysis

Scanning was performed on a 3T General Electric Signa PET-MR system with a 16-channel head coil. FDG diluted in saline was infused starting at time 0 of the PET-MR acquisition and continued for 60 min, with an initial radiation activity of ∼180 MBq distributed equally over the scan at a flow rate of 0.016 ml/s. Simultaneously acquired MRI sequences, starting from time 0, were in the following order: MRI attenuation correction, T1-weighted structural, T2-weighted FLAIR, and, at the 18 min mark, fMRI continuously for 42 min.

### Magnetic resonance imaging

Structural T1-weighted images were acquired for 7.36 min with the following acquisition parameters: FOV: 25 × 20 cm^2^, matrix: 256 × 256, slice thickness: 1 mm, slices: 180, TE: 3.1 ms, TR: 7200 ms, flip angle: 12, bandwidth: 244.1 Hz/pixel. The resulting voxel size was 0.49 × 0.49 × 1 mm^3^. T1 images were used for tissue segmentation (Ashburner and Friston, 2005), and normalization to standard MNI space (152 nonlinear sixth generation, MNI152NLin6Sym) using a preliminary 12 degrees of freedom registration with FMRIB's Linear Image Registration Tool followed by a nonlinear registration using FMRIB's Nonlinear Image Registration Tool (Jenkinson et al., 2012), resulting in 2 mm isotropic voxels.

#### fMRI

The BOLD data (sequence parameters: FOV: 25.6 cm, matrix: 96 × 96, slice thickness: 3.9 mm, TE: 30 ms, TR: 4000 ms, flip angle: 80°, acceleration factor: 2.0 using ASSET parallel imaging), with a voxel size of 1.95 × 1.95 × 3.9 mm^3^, were preprocessed with FSL FEAT (https://fsl.fmrib.ox.ac.uk/) (Jenkinson et al., 2012) and according to Stiernman et al. (2021). Briefly, this included motion correction by volume-wise rigid body transformation to the first volume, slice timing correction, spatial smoothing (FWHM 5 mm), and high-pass (120s) temporal filtering. First-level statistics were obtained using the GLM. The manipulation and maintenance conditions were either modeled as separate regressors across the entire experiment or as a single WM task (manipulation plus maintenance). Regressors of no interest were the extended motion parameters, 24 parameters, and framewise displacement. All resting periods (showing a fixation cross), including those within the embedded blocks, were treated as an implicit baseline. The main effects of WM over rest, and manipulation over maintenance, were the contrasts of interest. The resulting volumes were normalized to standard space using the T1 to MNI deformation fields, and a 6 degrees of freedom boundary-based registration between the first EPI volume and T1.

### PET

PET was dynamically acquired and reconstructed to 60 1 min frames using an ordered-subsets maximization algorithm with time-of-flight and point-spread function modeling [2 iterations, 28 subsets, and 6.4 mm post-filtering]. Correction for decay, scatter, and attenuation using an MR-based correction method was performed. The resulting voxel size was 0.97 × 0.97 × 2.81 mm^3^.

Following the approach we used in Stiernman et al. (2021), the PET data were motion-corrected, a Gaussian kernel [0.5, 1, 0.5] was applied to the data as a temporal filter, and was spatially smoothed (FWHM 8 mm). The mean activity between minutes 40-45 was skull-stripped and registered to the T1 image using FMRIB's Linear Image Registration Tool. Task-related changes in the slope of the time-activity curve (TAC) were analyzed using the GLM approach described previously (Villien et al., 2014; Hahn et al., 2016). Separately for each condition, task blocks were modeled as a ramp function with slope = 1 during the specific task and slope = 0 during rest and the other task. The task regressors were shifted forward by 2 min to account for the delay indicated by simulations performed previously (Stiernman et al., 2021). A baseline regressor was defined by a third-order polynomial across all gray matter voxels with the task regressors modeled as nuisance variables. The task regressors were then orthogonalized sequentially to the baseline regressor and added to the final GLM along with six nuisance movement regressors (*x*, *y*, *z*, yaw, pitch, roll). However, considering that the manipulation task also requires maintenance of information in WM; if added first into the orthogonalization and GLM, it may capture all common variance, despite orthogonalization. Accordingly, for the maintenance condition, we present statistics from a GLM with maintenance added first into orthogonalization, and for the manipulation condition with manipulation added first. The resulting β maps were normalized to MNI space using the T1 to MNI deformation field from the fMRI analysis pipeline. The main effects of WM over rest, as well as the contrast manipulation over maintenance, were the contrasts of interest.

Standardized uptake value ratio (SUVR) was calculated using the mean activity of the 24 first task-free minutes of the scan. The mean image had been preprocessed in the same manner as described in the previous section. The brainstem was used as reference. A SUVR value >1 indicated higher uptake in a voxel compared with the brainstem.

### Statistical analysis

FSL's randomize (Winkler et al., 2014) was used to perform higher-level analyses of both fMRI and fPET, using 10,000 permutations and treating a threshold-free cluster enhancement (TFCE) threshold of *p* < 0.05 as significant for a given voxel. Considering that the task paradigm used in the current study was selected based on previous studies showing robust task effects, no corrections for multiple comparisons were performed. To be inclusive in the presentation of the results, the standard threshold of reporting fMRI data was used (i.e., at *p* < 0.05, TFCE-corrected), along with uncorrected statistics. Voxelwise statistical analyses were projected to the cortical surface using the connectome workbench (https://www.humanconnectome.org/software/connectome-workbench). One-sample *t* tests were performed to derive the main effects of the task conditions (WM over rest, Manipulation over rest, and Maintenance over rest), and paired-sample *t* tests to compare Manipulation to Maintenance. In order to identify the metabolic basis of BOLD signal differences, the analyses of the change in glucose metabolism were restricted to masks including BOLD activations. Masks including all activated BOLD voxels for each of the main contrasts were produced (e.g., for the WM contrast); the mask included all activated fMRI voxels for both the young and old WM > rest contrast (i.e., activated BOLD voxels for the young WM > rest OR activated BOLD voxels for the old WM > rest). The manipulation over maintenance contrast was masked with an inclusive mask containing both WM over rest, Manipulation over rest, and Maintenance over rest, to prevent the exclusion of voxels particular to either condition. This mask was used to mask both the fMRI and PET analysis of the manipulation over maintenance contrast. Independent-sample *t* tests were then performed to compare the young and old groups on the main contrasts of interest and SUVR.

Conjunction overlap was calculated for the WM over rest, and manipulation over maintenance contrasts to test for activations common to both modalities and age groups. TFCE-corrected images thresholded at *p* < 0.05 were entered into the analysis and the following logical statement had to be true in order for a voxel to be considered as overlapping between both modalities and age groups (Nichols et al., 2005): Young fMRI AND Older fMRI AND Young fPET AND Older fPET. To further assess the correspondence between WM-induced changes in the BOLD signal and glucose metabolism, Pearson's correlations of the respective β estimates from the WM > Rest contrast were performed on the level of voxels in MATLAB version R2020b. To increase power, these correlations were computed across all individuals, and are reported in surface space as *r* values, using an uncorrected threshold of *p* < 0.05. To compare potential differences between young and old adults in terms of the scaling between BOLD and glucose metabolism, two additional analyses were performed. First, a linear regression was computed for each group, respectively, with WM > Rest β estimates from fPET predicting BOLD signal β estimates. This resulted in two separate betas: slope, signifying how much the BOLD signal increases for every unit increase in glucose metabolism. To test whether scaling differed between groups, a comparison of slopes was performed both on the level of voxels, and on the average estimates from the WM > Rest conjunction overlap mask (Young = 1 and Old = 2, β = slope, *n* = sample size).

$$t=\frac{\text{β}1-\text{β}2}{\sqrt{n1*sem1^{2}+n2*sem2^{2}}}$$

Using R statistics (https://R-project.org), independent-sample *t* tests were used to compare groups of young and old adults on demographic variables, SUVR in Figure 4*B*, and online and offline WM performance. When equality of variances could not be assumed, Welch's two-sample *t* test was performed. χ^2^ tests were used to compare sex differences. Finally, one-sample *t* tests were used to compare the offline WM performance of the older adults in the present study normalized to the performance of a population-drawn sample (Nordin et al., 2022) matched on age and education. A two-way analysis of variance was performed to test the age-related difference in the upregulation to WM demand in Figure 3*C*. For completion, the relation between WM accuracy and β estimates extracted from the WM > Rest conjunction overlap for BOLD and fPET was tested with linear regressions.

### Data availability

Group-level images and numerical source data that support the findings of this study are available at Open Science Framework (https://osf.io/rj4sm). The raw data from individuals are available from the Principal Investigator of the study on request, given appropriate ethical and data protection approvals.

## Results

### Cognitive performance

The in-scanner tasks entailed WM maintenance of four letters or WM manipulation of letters (moving maintained target letters ahead in the alphabet). The tasks were performed well by both age groups (Table 1). For the easier maintenance condition, the young and old groups performed at a comparable level, average accuracy >95%. For the more difficult manipulation condition, the young outperformed the older group, but mean accuracy was still high in both groups, >85%, and every participant performed above chance level. Together, the behavioral measures suggest that the in-scanner task was not taxing above capacity for either age group.

**Table 1.** Demographics and task performance

	Young (*n* = 23)	Old (*n* = 34)	*t* test*^a^*	Significance (*p*)*^b^*
Age (yr)	25.22 ± 4.02	71.09 ± 6.03	*t*_(55)_ = 31.937	<0.001
Sex (M/F)	10/13	17/17	χ^2^(1, *N* = 57) = 0.046	0.831
Education (yr)	15.23 ± 2.43	14.38 ± 2.73	*t*_(54)_ = −1.179	0.244
In-scanner tasks				
Manipulation (%)	92.97 ± 5.22	85.83 ± 10.06	*t*_(52.1)_ = −3.499	<0.001
Maintenance (%)	97.25 ± 3.00	95.44 ± 4.07	*t*_(55)_ = −1.818	0.075
Offline tasks				
WM (*z*)	0.72 ± 0.49	−0.50 ± 0.59	*t*_(55)_ = −8.214	<0.001
Letter updating (%)	85.69 ± 9.45	72.06 ± 11.85	*t*_(55)_ = −4.608	<0.001
n-back (%)	84.64 ± 3.49	66.32 ± 13.89	*t*_(35.4)_ = −7.025	<0.001
Spatial updating (%)	64.64 ± 18.36	36.08 ± 12.70	*t*_(55)_ = −6.951	<0.001

*^a^*Independent-samples *t* tests were used when equality of variances could be assumed, and Welch two-sample *t* test when not. A χ^2^ test was used to compare sex differences.

*^b^*Exact *p* values are reported down to *p* < 0.001.

### Task-dependent upregulation, common to both modalities and age groups

**Figure 1.**
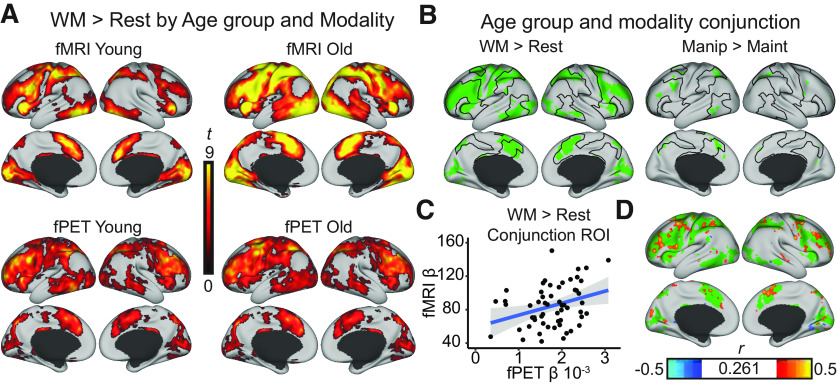
WM-induced activations for fMRI and fPET. ***A***, Activation maps for the WM over rest contrast are shown on the lateral surface from a dorsal view for each age group and modality. The activation maps were TFCE-corrected at *p* < 0.05 and projected to an inflated cortical surface using the human connectome workbench (https://www.humanconnectome.org/software/connectome-workbench). ***B***, The overlap between WM-induced BOLD activations and glucose metabolism was identified using TFCE-corrected maps at *p* < 0.05 for each age group and modality separately (i.e., the union of fMRI Young, fMRI Old, PET Young, and PET Old). The overlap of the four activation maps was then projected onto the cortical surface. The WM general analysis (Manipulation plus Maintenance tasks over rest) is displayed to the left. The upregulation to WM demand (Manipulation over Maintenance) is shown to the right (for the activation maps separated by age group and modality, see Fig. 3*A*,*B*). ***C***, Beta estimates of both modalities were extracted from the WM > Rest conjunction overlap map and plotted. Shaded area represents the Loess regression implemented in the *R* ggplot2 package. ***D***, Correlations were computed in each gray matter voxel and projected onto a cortical surface with the WM > Rest conjunction overlap map as underlay. The correlation map was thresholded at an uncorrected *p* < 0.05 (i.e., *r*_(57)_ > 0.261 or *r*_(57)_ < −0.261).

GLMs on the individual level, performed separately for both imaging modalities, were used to identify increases in the BOLD signal and glucose metabolism in response to WM (manipulation plus maintenance over rest, Fig. 1*A*) and WM demand (manipulation over maintenance).

An analysis of overlap of modality and group activation patterns showed that both age groups had overlapping fMRI and fPET activations in response to WM and WM demand (Fig. 1*B*) in the dorsal attention (DAN), ventral attention, and frontal-parietal control networks, highlighted with black lines based on an available functional cortical parcellation (Schaefer et al., 2018) based on the 7-network cortical parcellation (Yeo et al., 2011). General WM effects were widespread across these networks, but common upregulations of BOLD and metabolic activity in response to WM demand were most pronounced in middle frontal gyrus, superior frontal gyrus, paracingulate gyrus, superior lateral occipital cortex, superior parietal lobule, and inferior and middle temporal gyri (for specific peak locations, see Table 2). The patterns identified in the conjunction overlap are hereon referred to as “core WM areas.”

**Table 2.** Clusters from the conjunction of WM demand upregulation with the associated peak *t* value and location in MNI space [*X*, *Y*, *Z*] for each modality and age group*^a^*

	fMRI		PET	
Region	Young	Old	Young	Old
MFG/SFG	*t* = 6.92	*t* = 9.63	*t* = 5.35	*t* = 5.34
*k* = 617	[−30, 0, 58]	[−40, 4, 30]	[−26, 12, 46]	[−26, 18, 44]
Superior occipital	*t* = 10.2	*t* = 11.3	*t* = 7.08	*t* = 5.64
*k* = 194	[−32, −72, 48]	[−32, 76, 40]	[−24, −66, 38]	[−46, −54, 52]
Paracingulate *k* = 158	*t* = 5.77	*t* = 8.12	*t* = 3.82	*t* = 5.92
	[−4, 20, 44]	[−4, 16, 46]	[−2, 22, 40]	[−8, 20, 46]
SPL	*t* = 11.4	*t* = 10.8	*t* = 5.29	*t* = 6.05
*k* = 118	[−38, −54, 44]	[−30, −60, 40]	[−30, −60, 40]	[−32, −58, 46]
ITG/MTG	*t* = 4.48	*t* = 9.31	*t* = 3.27	*t* = 3.53
*k* = 101	[−54, −58, −6]	[−56, −54, −10]	[−52, −52, −10]	[−54, −50, −8]
SPL	*t* = 7.21	*t* = 9.22	*t* = 4.49	*t* = 3.83
*k* = 100	[34, −52, 40]	[40, −42, 46]	[34, −48, 42]	[34, −46, 46]

*^a^*Clusters with 100 adjacent voxels or more in gray matter with 0.50 probability are reported. ITG, Inferior temporal gyrus; *k*, cluster size; MFG, middle frontal gyrus; SFG, superior frontal gyrus; MTG, middle temporal gyrus; SPL, superior parietal lobule.

Across the sample, β estimates extracted from the WM conjunction overlap were positively correlated: *r*_(55)_ = 0.321, *p* = 0.015 (Fig. 1*C*), *r*_(21)_ = 0.087, *p* = 0.693 for the young adults, and *r*_(32)_ = 0.413, *p* = 0.015 for the old adults. Areas where positive voxelwise correlations arise are shown in Figure 1*D* and generally fall within the higher-order association areas identified in Figure 1*A*, including dorsolateral PFC, anterior cingulate, and superior parietal cortex. The slope for the association between fPET and fMRI betas did not differ between age groups (*t*_(53)_ = −0.231, *p* = 0.818).

### BOLD fMRI overactivation outside core WM areas is not accompanied by change in glucose metabolism

**Figure 2.**
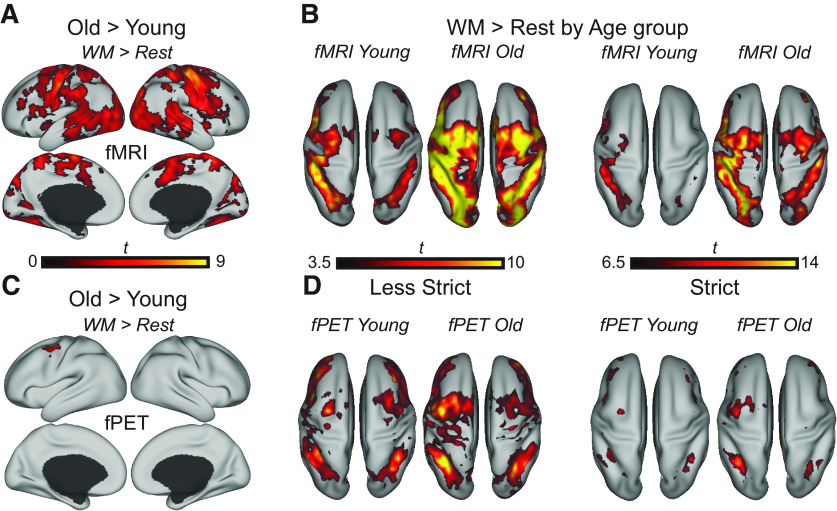
Age-related upregulation of fMRI BOLD and glucose metabolism during WM. ***A***, Age-related differences were detected only for the old over young contrasts. The WM general analysis (Manipulation plus Maintenance tasks over rest) of age on fMRI BOLD is displayed on the cortical surface. ***B***, The WM general fMRI analysis at a less strict threshold (TFCE *p* < 0.05 and *t* > 3.5) is shown on the left, and at a stricter threshold (TFCE *p* < 0.05 and *t* > 6.5) to the right. ***C***, The WM general analysis of age on fPET glucose metabolism is displayed on the cortical surface. ***D***, The WM general fPET analysis at a less strict threshold (TFCE *p* < 0.05 and *t* > 3.5) is shown on the left, and at a stricter threshold (TFCE *p* < 0.05 and *t* > 6.5) to the right.

Although activity patterns in core WM areas converged between age groups based on a minimum threshold (previous section), the BOLD signal of older adults was more widespread and thus significantly higher in regions primarily outside core WM areas (Fig. 2*A*). Framewise displacement, estimated from fMRI, was unrelated to the mean β estimates, extracted from Figure 2*A*, of both fMRI and fPET in the young and old adults (all *p* > 0.29, and the range of *r* values was −0.227 to 0.102). To better understand patterns of age-related BOLD signal differences, the main effects of WM (> rest) are displayed at one less strict and one stricter threshold in Figure 2*B* from a dorsal view. As the threshold is made stricter (shown with *t* > 6.5, *p* < 0.000001), it can be appreciated that older adults show more bilateral recruitment of frontal and posterior regions in terms of BOLD, not visible for the young group at this threshold; this pattern is highly consistent with age-related reduction in hemispheric asymmetry (Cabeza, 2002; Schneider-Garces et al., 2010). Conversely, the less strict threshold (TFCE-corrected at *p* < 0.05, and an uncorrected threshold akin to *p* < 0.001 for the young group) suggests that a more diffuse bilateral pattern is, at least in part, reflective of an overall higher signal in older adults that extends beyond canonical network borders.

Crucially, neither reduced hemispheric asymmetry of core WM areas nor a more widespread pattern extending into neighboring networks was detected in voxelwise comparisons of glucose metabolism (Fig. 2*C*,*D*). One exception for age-related differences in glucose metabolism is a focal increase in older adults in left premotor cortex/frontal eye fields, located primarily within the DAN. Importantly, age-related differences in WM-related glucose metabolism did not appear robust outside the DAN even at an uncorrected threshold of *p* < 0.05 (data not shown), suggesting that differences between modalities are not simply because of a statistical thresholding issue.

**Figure 3.**
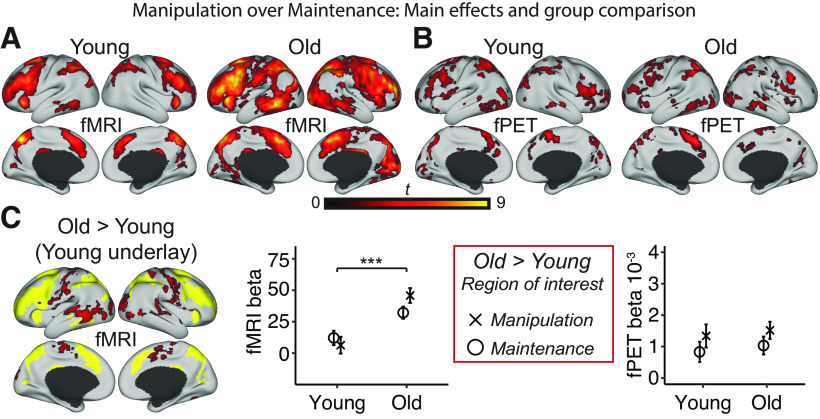
Age-related upregulation to WM demand. ***A***, The BOLD signal upregulation to WM demand (Manipulation over Maintenance) is shown for the young and old adults separately. ***B***, The corresponding activation maps for fPET are displayed. ***C***, The age-related upregulation to WM demand is shown only for fMRI BOLD as no significant areas of activation was observed with fPET. Significant age-related differences in the upregulation to WM demand is overlaid on the main effect of WM demand for the young (shown in yellow, i.e., a binarized map from ***A***), highlighting regions on the network borders where older adults show more widespread activations. The mean betas in all activated voxels from the Manipulation over Maintenance and Old over Young contrast were extracted and plotted for each age group and modality separately. Error bars indicate 95% CI. ***p < 0.05, significant age × condition interaction. All surface projections included activations significant at a corrected TFCE *p* < 0.05.

Activation maps for the upregulation to WM demand are shown in Figure 3*A*, *B*. Age-related upregulation of BOLD activity in response to WM demand was also found to be higher in the old (Fig. 3*C*). Conversely, there was again no age-related upregulation to WM demand in terms of glucose metabolism. Age-related upregulation WM demand was located primarily on the borders of the areas of increased activation in the young adults (Fig. 3*C*, yellow underlay). The regions differing in this age group × difficulty interaction largely resembles those reported in a previous study investigating load effects on a visuospatial WM task between similar age groups (Jamadar, 2020). There were no regions displaying increased upregulation for the young compared with the old group either in terms of BOLD or glucose metabolism.

### Glucose hypometabolism in older adults

**Figure 4.**
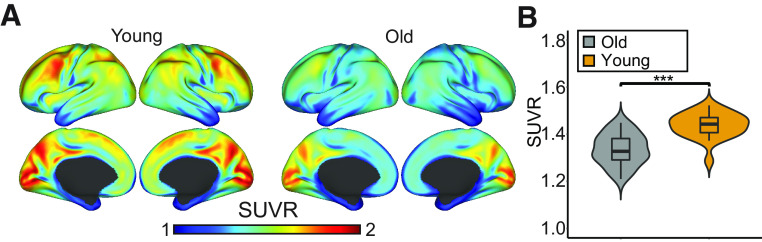
Relative glucose hypometabolism in older adults. ***A***, SUVR was calculated with the brainstem as reference and projected onto an inflated surface. The color bar intensity range was set to SUVR values ranging from 1 to 2. ***B***, Violin plot represents the distribution of values and difference between young and old adults SUVR values averaged over all gray matter voxels in the brain. Thick line in the violin plot indicates the median SUVR, hinges depict the 25th and 75th percentiles, and whiskers the smallest/largest observation greater/less than the hinge plus 1.5 times the interquartile range. ***p < 0.001.

As all GLM analyses focus on relative changes in activation, SUVRs were computed as a semiquantitative indicator of baseline glucose metabolism in each individual from the first 24 min of PET data (i.e., before any task started). Figure 4*A* shows that older adults had lower glucose metabolism than younger adults across the cortex. A statistical comparison with a TFCE (*p* < 0.05) revealed that SUVR in the old was significantly lower in 68% of all gray matter voxels in the brain, and significantly higher in only 1%. The average SUVR across all gray matter voxels in the brain was significantly different; mean young = 1.44 ± 0.05, mean old = 1.34 ± 0.07, *t*_(55)_ = 6.27, *p* < 0.001. Hence, older adults were able to upregulate glucose metabolism in response to WM like the young, despite displaying lower glucose metabolism across the brain.

### Comparison of the older adults WM function to a population drawn sample

Younger and older adults performed an offline (out of the scanner) cognitive test battery to yield a composite score for WM. Both groups were highly educated, but younger adults had significantly higher performance scores than older adults on all measures (Table 1). Nevertheless, when comparing the older sample from this study, that were recruited by newspaper ads, to an age- and education-matched comparison sample drawn from the population in the same area (Nordin et al., 2022), we found that our study sample performed 0.44 SD higher on the WM composite (*t*_(33)_ = 3.628, *p* < 0.001). This analysis shows that the results presented on age differences in the current sample should be interpreted in light of having a high performing sample of older adults.

For completeness, we here assess the relation between WM performance and WM-induced changes in BOLD and glucose metabolism, primarily to test whether overactivations may yield performance benefits. Linear regressions were performed to test the association between average WM accuracy for the in-scanner tasks and β estimates extracted from the WM > Rest conjunction overlap for both modalities. WM accuracy was unrelated to BOLD in the young group (*R*^2^ = 0.046, *F*_(1,21)_ = 1.02, *p* = 0.324) and to fPET (*R*^2^ = 0.110, *F*_(1,21)_ = 2.607, *p* = 0.121) and to BOLD in the old group (*R*^2^ = 0.001, *F*_(1,32)_ = 0.038, *p* = 0.847) and fPET (*R*^2^ < 0.001, *F*_(1,32)_ < 0.001, *p* = 0.996). Controlling for age did not influence the results. In the absence of notable performance differences within individuals of either group, these results should be interpreted with caution.

## Discussion

In this study, we used simultaneous fPET/fMRI to investigate whether commonly observed age-related BOLD overactivations with intact WM performance are concomitant with age-related increases in glucose metabolism in response to WM tasks. Glucose metabolism, as assessed with FDG fPET, complements the BOLD signal since it provides a marker of synaptic activity that is independent of blood flow (Villien et al., 2014). fPET is therefore of importance to establish whether BOLD overactivations are indicative of increased neural activity.

Two main findings emerged. First, consistent with the view that both modalities are sensitive to increased metabolic demands in response to neural firing, we find that WM and WM demand-related changes converge between BOLD and glucose metabolism in a set of regions involved with cognitive control and attentional processing (Fig. 1*B*). Second, and of critical importance, age-related increases in the WM-related BOLD response were not matched by corresponding increases in glucose metabolism, suggesting that commonly observed overactivations with aging are not neuronal in origin.

### No evidence for functional reorganization with age

Prior work has suggested that overactivation in older adults, in combination with good task performance, could reflect the fact that older adults expend greater brain activity to counteract age-related deficits in neural resources (Cabeza et al., 2018). In the current study, fMRI activation patterns showed typical pattens of overactivation in task-related areas, consistent with other age-comparative WM studies (e.g., Cappell et al., 2010; Schneider-Garces et al., 2010; Spreng et al., 2010). However, in the fPET data, we found little evidence for increased glucose metabolism in the regions of overactivations. Given that FDG is sensitive to synaptic activity (Kadekaro et al., 1985) but insensitive to changes in blood flow (Villien et al., 2014), our results strongly suggest that overactivations in the older adults are not neuronal in origin, and thus unlikely to indicate a neuronal reorganization or selection of unused compensatory neural resources in an aging brain (for a similar critique, see Morcom and Henson, 2018). To further illustrate these patterns, at lower statistical thresholds, older adults showed considerably more diffuse BOLD activation patterns compared with young adults and to fPET (Fig. 2*B*,*D*). At a more stringent threshold, older adults showed bilateral recruitment of frontal and parietal regions, displaying a typical pattern of hemispheric asymmetry reduction (Cabeza, 2002). However, considering that this pattern was only evident at a more stringent threshold, it is possible that evidence for reorganization may rather be a question of statistical thresholding, and upregulation of BOLD signal, than reorganization per se.

Older adults exhibited higher glucose metabolism during WM only in an isolated cluster in left frontal eye field/premotor cortex. This may indicate greater need for top-down control over visual areas in the older adults (Vossel et al., 2014), and/or alternatively, pointing to greater demand for fine motor skills or response selection on older adults (Seidler et al., 2010). Considering that only contralateral motor regions displayed age-related increases in glucose metabolism (Fig. 2*C*), the suggestion that compensatory recruitment of ipsilateral motor regions are neuronal (Seidler et al., 2010; Turesky et al., 2016) is not supported in the current study (for a similar critique, see Knights et al., 2021).

### Potential mechanisms behind altered neurovascular coupling that leads to overactivations

If elevated BOLD responses in regions that are not recruited by younger adults, or recruited to a lesser extent, are not reflective of neuronal activity in older adults, it is of critical importance to uncover what overactivations reflect. Unlike the fPET signal, the BOLD signal depends on neurovascular coupling, which, when coupled, reflects changes in local blood oxygen concentrations in response to neural activity (Logothetis et al., 2001). Most notably, age-related changes in cerebral vasculature affect the structure and function of arterial walls, which affect the blood flow itself, and consequently may affect the HRF magnitude (D'Esposito et al., 2003), as well as its shape and timing (West et al., 2019; Krishnamurthy et al., 2020).

It is then possible that a lower baseline BOLD signal in older adults (Krishnamurthy et al., 2020) may cause relatively larger stimulus-evoked changes in the BOLD signal (Cohen et al., 2002; Lu et al., 2008; Tsvetanov et al., 2021), giving rise to BOLD overactivations in absence of corresponding increases in glucose metabolism. Nevertheless, we think that altered vasculature does not readily explain overactivations in healthy, older adults. To reiterate, there is little evidence for cognition-enhancing longitudinal increases in fMRI signal during cognitive tasks (Hakun et al., 2015; Rieckmann et al., 2017; Pudas et al., 2018; Vaqué-Alcázar et al., 2020), whereas there are robust longitudinal changes in vascular functions in old age. Moreover, a potential mechanism for overactivations should be reconcilable with common observations of overactivations (Nyberg et al., 2014; Cabeza et al., 2018) and WM load-dependent upregulation (Nagel et al., 2009; Kennedy et al., 2017; Salami et al., 2018) with better cognitive performance, whereas vascular pathology is more likely to be found in poor performers.

Other potential mechanisms for explaining altered neurovascular coupling that could underlie overactivations, in the absence of increased glucose metabolism, concern neuromodulatory systems. In macaques, a systemic increase of dopamine disproportionally increased metabolic demands but decreased the BOLD response (Zaldivar et al., 2014). This suggests that the relation between a dynamic increase in metabolism (in our case, by a cognitive task) and the corresponding BOLD response is modulated by dopamine. If these findings translate to individual differences in dopamine functions, then for individuals with higher dopamine release (i.e., younger adults), high metabolic demand should be accompanied by a comparatively lower BOLD signal. In line, our previous work found a greater and less differentiated pattern of frontal fMRI coupling in younger adults during task performance when they were given a D1 receptor antagonist (Rieckmann et al., 2012).

Another catecholamine, norepinephrine, has been shown to optimize the oversupply of blood in response to increases in metabolism in mice (Bekar et al., 2012). Since the BOLD signal is dependent on the oversupply of blood, lower levels of norepinephrine in older adults might result in a generally less focused BOLD signal (i.e., more widespread and higher; Fig. 2*A*,*B*). To our knowledge, there are no studies investigating norepinephrine levels during the aging process in humans, but age-related decreases in norepinephrine has been shown in the rat (Míguez et al., 1999), and a linear reduction in norepinephrine transporter levels has been observed from young adulthood to late mid-life in healthy humans (Ding et al., 2010). Since dopamine and norepinephrine not only affect neurovascular coupling but also cognitive performance via other mechanisms, the precise contributions of individual differences in neurotransmitter tone to overactivations in older adults remains a topic for future research.

### Limitations

The focus of the current study was on overactivations that are observed when older adults perform within their WM capacity (i.e., when performing a task well). When task demands are high enough that the task performance of older adults approach chance level, convergent upregulation of BOLD signal and glucose metabolism in core WM areas should fail and result in a decrease of both signals for older adults. Such observations are common with BOLD, for example, during a 3-back task (Mattay et al., 2006; Nyberg et al., 2009) or increasing maintenance set sizes (Cappell et al., 2010; but see Jamadar, 2020). In the current study, we did not investigate brain activation patterns across many conditions of WM load as the fPET temporal resolution limits the number of different task conditions. Future studies with different tasks and task demands are needed to corroborate our findings further. It should be noted that the embedded design used in the current study differs from what is typically seen in fMRI experiments (e.g., in terms of the long duration of rest blocks), which may affect the β estimates used for analyses of individual differences. Furthermore, the design prevents detailed analyses of the shape and timing of the HRF (West et al., 2019). However, on the group level, typical patterns of age-related overactivations were observed, and we believe the general conclusions regarding group level effects are still valid. With future advances in PET methodology, hybrid task-related activation studies should aim to better align the temporal resolution of both modalities to isolated events or short task blocks (Rischka et al., 2018).

In conclusion, WM and WM demand-related changes in the vascular response estimated with fMRI BOLD, and synaptic activity estimated with fPET, converge in core WM regions in young and older adults alike. This finding is in line with theories asserting that high-performing older adults should display similar activation patterns as younger adults (Nyberg et al., 2012). Conversely, findings from the current study show that the overactivations observed both within and outside core WM regions with fMRI in old adults are not coupled with increased glucose metabolism, which suggests that the overactivations are not neuronal in origin. Future research should investigate alternative physiological mechanisms, for example, whether differences in neurotransmitter tone modulate neurovascular coupling differences in extreme age group designs.
